# Application of extrusion-cooking technology in hatchery waste management

**DOI:** 10.1515/biol-2020-0065

**Published:** 2020-08-13

**Authors:** Aldona Sobota, Piotr Zarzycki, Anna Wirkijowska, Zbigniew Rzedzicki, Agnieszka Pawlas

**Affiliations:** Division of Engineering and Cereals Technology, Department of Plant Food Technology and Gastronomy, Faculty of Food Science and Biotechnology, University of Life Sciences in Lublin, 8 Skromna Street, 20-704 Lublin, Poland

**Keywords:** chemical composition, corn meal, extruded feed, physical properties, microstructure

## Abstract

The purpose of the present study was to examine the extrusion-cooking process of cereal blends with addition of hatchery waste (HW). The effect of HW addition on the physical properties and chemical composition of the extrudates was examined. The share of the HW in blends with corn meal was variable in the range of 5–30%. The study was conducted using a counter-rotating twin-screw extruder (L:D ratio 12:1, screw speed 75 rpm, die diameters 3 mm × 6 mm, profile of barrel temperature 120/140/180/180/130°C, and material moisture 28%). Increase in the content of the HW from 5 to 30% reduced the expansion ratio, pellet durability index, water absorption index, and water solubility index of the extrudates. The microstructure of the extrudates with HW addition is non-homogeneous; the areas with densely packed and adherent granules, interwoven with fibrous structures, can be observed. Increase in the share of HW leads to a lowering of the levels of crude fiber and total dietary fiber in the extrudates. Moreover, a significant increase in the content of protein, crude fat, and mineral elements such as calcium, sodium, magnesium, and iron was observed.

## 1 Introduction

In recent years, the constantly growing demand for poultry products, which led to an intensive development of the hatchery industry and the associated production of hatchery waste (HW), has given rise to a serious environmental problem [1,2]. An improper disposal of the poultry waste may lead to contamination or degradation of the environment and surrounding ecosystems [1,2,3]. The HW comprises empty shells, infertile eggs, dead embryos, late hatchings, dead chickens, and a viscous liquid from eggs and decaying tissues [4]. The HW is mostly utilized as compost, incinerated, or processed into the biogas [5]. However, potentially, it is a valuable feed material, rich in proteins, fat, and minerals (calcium in particular) [1,2,6,7,8,9]. Currently, hatchery by-products are allowed in many countries including the United States and the European Union in animal feeds [4,10]. Nutrition studies revealed that properly processed HW can be a cheaper and very good substitute for animal protein sources such as fish meal and spray-dried plasma protein in animal feeds [1,2,5,9,11]. However, the high moisture content (MC) of the wastes and the specificity and presence of numerous microorganisms make it a highly unstable material. Its utilization, transport, and processing at poultry hatcheries pose considerable problems. They are mostly used to produce hatchery meal [12]. Wastes rendered by cooking, drying, and grinding into a meal-hatchery by-product are pathogen-free [7,9]. An alternative and simpler method of processing HW is the extrusion-cooking technology. The application of suitable extrusion parameters (temperature, material retention time, and MC of raw material) permits us to achieve a totally sterile product with attractive physicochemical properties and high nutritional value [12,13,14,15,16,17]. Okelo et al. [14] demonstrated that under moderate extrusion conditions (MC 28.5%, barrel temperature 83°C, and retention time 7 s) *Salmonella typhimurium* was eliminated from feed. Similar results were obtained by other authors [13]. Extrusion was most effective in reducing the total coliform count in HW, as compared to cooked and autoclaved methods [15]. The twin-screw extrusion guarantees sterility of the extrudate, if a minimum time of material retention in the extruder is guaranteed for a given temperature. The most thermoresistant strains of bacteria (*Bacillus stearothermophilus FS 1518*) were sterilized at an extrusion temperature of 182°C and a material retention time of 45 s [18].

The purpose of the present study was to examine the possibility of processing HW with the method of extrusion and the effect of the addition rate regarding the waste component on the physical properties and chemical composition of the extrudates.

## 2 Materials and methods

### 2.1 Raw materials

The materials used in this study were HW, acquired from the poultry hatchery Malec (Dębówka, Poland), and corn meal (Agropol, Motycz, Poland). The chemical composition of the materials used in the study is presented in Table 1. The HW was incorporated into recipes at replacement levels for corn meal at levels (w/w) of 5% (HW5), 10% (HW10), 15% (HW15), 20% (HW20), 25% (HW25), and 30% (HW30). The ingredients were moistened to 28% moisture and mixed in a drum mixer type H-095 (Agro-Wikt, Opoczno, Poland). Material moisture of 28% permitted the process of “dry” extrusion.

**Table 1:** The content of dry matter (% w.b.) and chemical components (% d.b.) of raw materials

Chemical components	Corn meal	Hatchery waste
Dry matter	86.50	58.82
Protein	11.20	14.19
Crude fat	4.11	8.52
Ash	1.71	73.22
NDF	12.36	—
ADF	6.47	—
Hemicelluloses^a^	5.89	—
Cellulose^b^	5.37	—
Lignin	1.10	—
TDF^c^	12.33	—
IDF	9.81	—
SDF	2.52	—

NDF – neutral detergent fiber; ADF – acid detergent fiber; TDF – total dietary fiber; IDF – insoluble dietary fiber; SDF – soluble dietary fiber; ^a^Hemicelluloses = NDF–ADF; ^b^Cellulose = ADF–Lignin; ^c^TDF = IDF + SDF; hemicellulose, cellulose, and TDF contents were calculated from replicates.

### 2.2 Extrusion parameters

The study was conducted using a counter-rotating twin-screw extruder (Metalchem, Gliwice, Poland) with conical screws and an L:D ratio of 12:1. The screw speed applied was 75 rpm and die diameters were 3 mm × 6 mm. Retention time of the mass in the extruder chamber was 90 s ± 10. The profile of temperature distribution in the extrusion-cooker barrel was 120/140/180/180/130°C. The process parameters applied in the study and the composition of mixtures were determined from pilot experiments. Only such parameter ranges that guaranteed correct and stable run of the process were adopted.

### 2.3 Analysis of physical properties

Expansion ratio (ER) was calculated as the ratio of the cross-sectional area of the extrudates to the cross-sectional area of the die. Pellet durability index (PDI) of the extrudates was determined following ASAE Method No. S269.4 [19]. Approximately 500 g of extrudates from each blend was manually sieved (mesh size 3.4 mm) for about 10 s to remove initial fines and then tumbled in a pellet durability tester (Model PDT-110; Seedburo Equipment Company, Chicago, USA) for 10 min. Later, the samples were again hand sieved (mesh size 3.4 mm) for about 10 s and then weighed. PDI was calculated by the following equation:

\text{PDI}\hspace{.25em}( \% )=({M}_{\text{a}}/{M}_{\text{b}})\times 100,

where *M*_a_ is the mass of the extrudates retained on the screen after tumbling (g) and *M*_b_ is the mass of the extrudates retained on the screen before tumbling (g). The water absorption index (WAI) and water solubility index (WSI) of the extrudates were assayed with the centrifuge AACC method 88-04 [20].

### 2.4 Scanning electron microscopy

The microstructure of extrudates was studied in a sample with 20% HW content. The sample was sliced into fragments that were then glued with silver paste onto specimen circles and sprayed with carbon and gold in a vacuum sprayer-type JEE 4X (JEOL, Tokyo, Japan). Microscopy analyses were carried out via an electron microscope of the type JSM 5200 (JEOL, Tokyo, Japan).

### 2.5 Chemical analysis

MC was determined using the AACC method 44-15A [20]. The samples were dried at 105°C to constant weight in a gravity-convection oven (Wamed, Warsaw, Poland). The ash content was assayed according to the AACC method 08-01 [20], protein content with the AACC method 46-08 [20], using the nitrogen to protein conversion factor of *N* × 6.25. The content of the detergent fractions of dietary fiber in the samples was found to conform to that of the method developed by Van Soest [21,22]. The content of neutral detergent fiber (NDF) was assayed without heat stable amylase and expressed inclusive of residual ash.

Enzymatic method was applied to determine the content of total dietary fiber (TDF), insoluble dietary fiber (IDF), and soluble dietary fiber (SDF) fractions. In the determination of enzymatic fiber, Megazyme enzymes and methodological procedures were employed according to AOAC (methods 991.43 and 985.29) and AACC (methods 32-07, 32-21, and 32-05) [20,23]. The content of minerals (calcium, magnesium, sodium, potassium, copper, iron, zinc, chromium, and nickel) in the extrudates was assayed using the AOAC method 975.03 [23].

### 2.6 Statistical analysis

Chemical analyses were carried out in three replications, and physical properties were analyzed in five replications. Mean values and pooled standard error of the means were calculated. The significance of differences among the results was determined using the Duncan test (*P* < 0.05). Regression models describing the correlation between physical properties of extrudates and percentage share of HW were developed. The statistical analysis of the results was performed using the program SAS 9.1.3 (SAS Institute Inc., Cary, NC, USA).

## 3 Results and discussion

The application of counter-rotating twin-screw extrusion permitted the processing of plant-HW blends with a maximum 30% content of the waste component. Any higher content of HW led to caking on the cylinder walls and on the screws and blocking of the extruder. A slightly lower level of HW in a blend with corn meal was applied by Miller [24], who extruded blends comprising 25% HW and 75% ground corn. In this study, the material was extruded at a constant moisture of 28%. The moisture level applied permitted dry extrusion, while retaining a high nutritional value of the product. According to Rokey [25], the optimum moisture level for processing feed blends is 25–30%. Lower moisture levels during extrusion contribute to the destruction of heat-labile nutrients such as lysine and ascorbic acid [25]. The pilot study and the research conducted by Zarzycki et al. [16] demonstrated that at a blend moisture of above 28%, secondary agglomeration of the material occurs, with the material sticking to the feeder and extruder screws. The disturbance in the feeding of the material resulted in reduced efficiency and extended time of material retention in the extruder, and in consequence to caking of the material on extruder cylinder and screws and to destabilization of the process. Moreover, blend moisture at the level of 28% makes it possible to use waste component with even a considerably higher moisture compared to the waste material used in this study. Miller [24] observed that extrusion causes a notable reduction of material moisture, facilitating the turnover and storage of feeds containing HW, which finds support in this study. The moisture of the products with a content of HW did not exceed 9% (Table 3). According to Ah-Hen et al. [26], extruded feed with moisture below 12% has water activity on a safe level and does not require extra drying after extrusion.

Extrusion-cooking with a counter-rotating twin-screw extruder permitted the processing of blends with up to 30% of HW content. Increase in the content of the waste component caused a decrease of ER and of the PDI of the products (Table 2). At the same time, products with 30% of HW content were characterized by notably lower WAI and lower WSI, compared to extrudates with the lowest, 5% content of the waste (Table 2).

**Table 2:** Physical properties of the extrudates

	Samples					
	HW5	HW10	HW15	HW20	HW25	HW30
ER	2.89^a^	2.62^b^	2.59^b^	2.50^c^	2.50^c^	2.47^d^
PDI (%)	87.91^a^	89.38^a^	81.40^b^	75.52^c^	72.16^d^	68.18^e^
WAI (%)	462.05^a^	374.47^b^	399.75^b^	329.87^c^	320.73^c^	322.40^c^
WSI (% d.b.)	5.58^a^	5.36^b^	5.04^c^	5.15^c^	4.72^d^	4.63^d^

Mean values with different letters (a–e) in the same row are significantly different (*P* < 0.05); ER – expansion ratio; PDI – pellet durability index; WAI – water absorption index; WSI – water solubility index; HW – hatchery waste.

**Figure 1:**
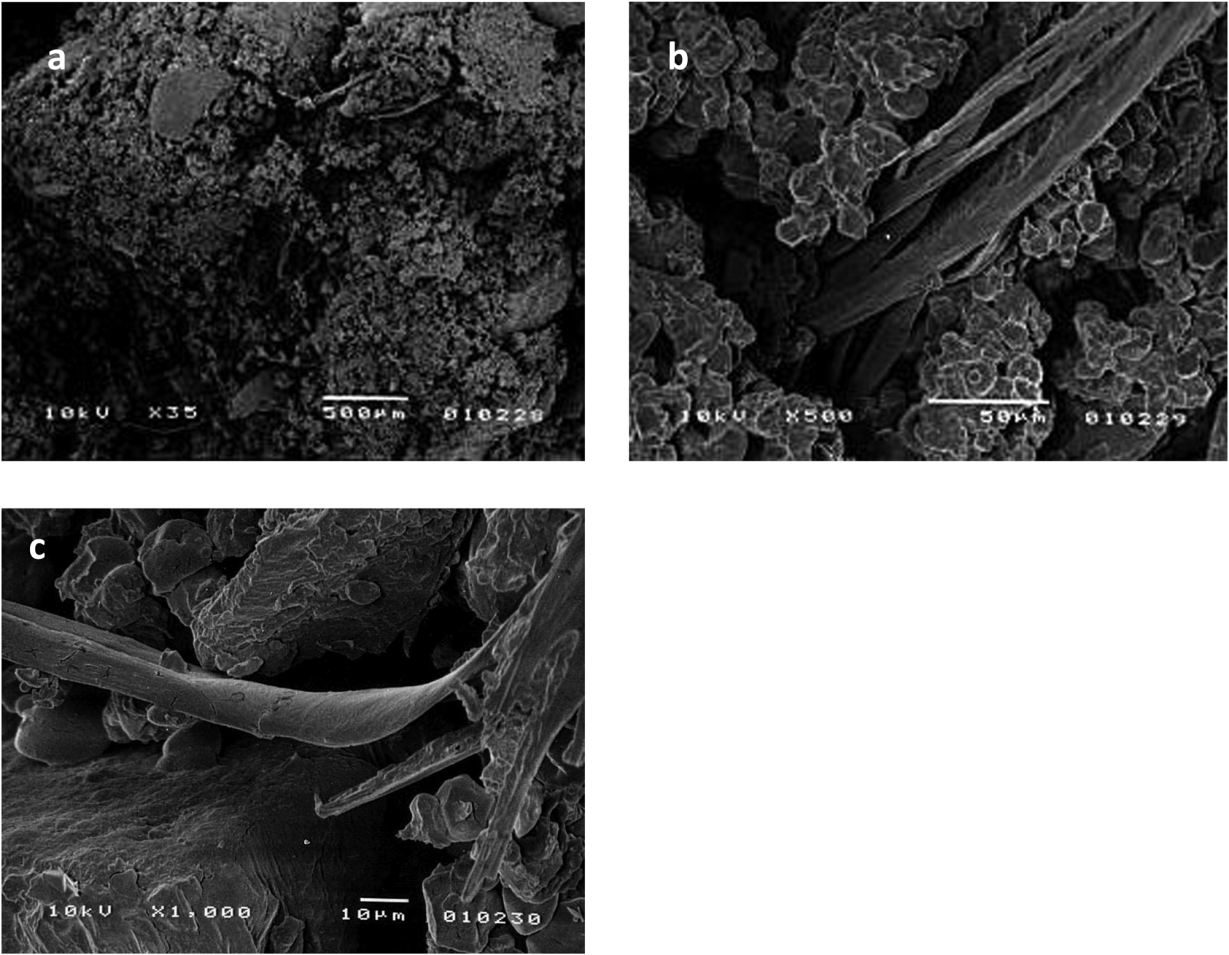
Scanning electron macro- and micrographs of extruded products. Raw composition: 20% hatchery waste and 80% corn grits (HW20). Magnification: (a) ×35; (b) ×500; and (c) ×1,000.

When analyzing the physical properties of animal feeds, it should be borne in mind that different animal species require different physical properties (particle size, hardness, PDI, WAI, and WSI) for their respective feeds. This means that different quality standards are used to evaluate the feed products [27]. Products with a 5–30% of HW content were characterized by a bright color and low expanded, compact structure. The observed microstructure of extrudate with 20% of HW content (Figure 1) is non-homogeneous and differs decidedly from the microstructure of typical high-starch extruded products [28,29]. There is no cellular structure characteristic of expanded products; however, areas with densely packed and adherent granules, interwoven with fibrous structures, can be observed. It appears that the elements of corn endosperm, liquefied more easily in the course of the process, acted as a specific adhesive for the less easily liquefied fragments of HW. The fibrous structures observed are non-liquefied fragments of chicken feathers.

Increase in the content of the waste component led to a decrease in the degree of ER. The above correlation can be described using the formula for the regression (*R* ^2^ = 0.83):

\text{ER}=0.0009{\text{HW}}^{2}\mbox{--}0.0465\text{HW}+3.052.

The decrease in ER on increasing the share of HW results probably from the lowering of share of corn meal in a mixture. In the opinion of numerous authors, protein content has a significant effect on the radial ER. Seker [30] claims that the formation of a strong protein matrix can inhibit the liberation of water vapor from the product exiting the die of the extruder and contribute to the formation of a compact, less expanded structure of extrudates.

The PDI represents an important parameter in the evaluation of extrudates and it expresses the resistance of products to crumbling. Ayadi et al. [31] and Samuelsen et al. [32] emphasize that extruded feeds should be characterized by high durability in transport and storage. However, when using extruded products as a component of feed blends, too high a mechanical strength is associated with higher energy consumption during the grinding and may cause problems in achieving an adequate degree of fragmentation. PDI of extrudates with HW ranged from 68.18% to 87.91% (for samples with 30% and 5% share of HW, respectively). Correlation between PDI and the share of HW is described by the formula of regression (*R* ^2^ = 0.94):

\text{PDI}=-0.8922\text{HW}+94.704.

It can be observed that the mechanical strength of the products is reduced on increasing the content of HW, which may be a result of changes to the microstructure. The non-homogeneity of the structure of extrudates with 20% of HW content (Figure 1) was most probably amplified because of increasing content of the less easily liquefied waste component. This could have led to a reduction in the mechanical strength of the products. According to Ayadi et al. [31], the chemical composition of extruded blends and contents of ingredients such as protein, fat, fiber, and minerals can have an important influence on the PDI value. The decrease of PDI with an increase of HW share could be attributed to the increase in fat and the concomitant decrease in starch content in the raw ingredient blends. Fat contained in the HW component could have caused a lubricating effect and thus decrease the shear rate gradient, lowering the degree of processing of the material.

WSI is an especially important parameter in the case of fish feed. The products of this type should be characterized by high water stability and possible low values of WSI [33]. Increase in the content of HW caused a reduction in the value of WSI. This correlation is described by the formula of regression (*R* ^2^ = 0.92):

\text{WSI}=\mbox{--}0.0375\text{HW}+5.736.

The decrease in WSI on increasing the share of HW results probably from the decrease in the amount of component with high-starch content. The relationship observed supports literature data. Significant effect on WSI extrudates can also have the content of fat in raw materials. Fat acting as grease causes a decrease in the shear rate gradient in an extruder and influences the lower degree of depolymerization of starch. Sobota and Rzedzicki [34] reported that the value of WSI of extrudates is a measure of the degree of degradation of the molecular mass of starch polymers and at the same time provides information on the degree of processing of extruded products. The water solubility of polysaccharides increases with a decrease in their molecular weight. WAI is also important in fish food that is characterized by considerable resistance to hydration and soaking in a water environment. The extrudates studied were characterized by a relatively low WAI, ranging from 462.05% to 322.4% (Table 2). Increase in the content of HW and the concomitant decrease in the level of the high-starch component (corn meal) resulted in a decrease of water absorption of the extrudates. This correlation is described by the formula of regression (*R* ^2^ = 0.82):

\text{WAI}=0.3275{\text{HW}}^{2}-15.918\text{HW}+527.57.

According to many authors, the WAI of extruded products is positively correlated with the degree of damage to starch granules [31]. A higher content of the fat-rich waste component could reduce the friction, thus slowing down the damage to the starch granules. Ayadi et al. [31] emphasize that the chemical composition of products has a strong influence on the WAI. In the opinion of Fernandez-Gutierrez et al. [35], the lower hydration capacity observed with an increase in protein content may be caused by the formation of intramolecular bonds between protein and starch polysaccharides (amylose and amylopectin).

The chemical composition and nutritional value of HW highly varied and depend on the hatching rates of poultry and on the variable amounts of shells, eggs, embryos, and culled chicks [6,8,12]. This makes the discussion on comparison between the obtained results and results of studies by other authors difficult. The wastes used in this study were characterized by very high ash content (73.22 % d.b.), and at the same time relatively low moisture (41.18%) and low content of protein (14.19% d.b.) and crude fat (8.52% d.b.). This indicates that egg shells constituted a considerable component in the waste material. According to Glatz et al. [12], HW is a high protein material with 43–71% moisture. Dried HW contains 33.1% crude protein, 29% crude fat, 12.1% crude fiber, and 21.5% ash. A different chemical composition of HW was reported by Al-Harthi et al. [7] and Sathishkumar and Prabakaran [9]. These authors noted a considerably lower content of crude fiber (1.2% and 0.92%, respectively) and a higher content of protein (36.5% and 36.24%, respectively).

The HW used in the study was a very rich source of minerals (Table 1). Their content assayed in the form of ash amounted to 73.22% d.b. The increase in the content of HW from 5 to 30% caused a more than fourfold increase of ash content in the products (Table 3). Upon analyzing the levels of selected minerals in the function of increasing HW content, an especially large increase was noted in the content of calcium (Table 4). Extrudates with 5% addition of HW contained 12.639 g/kg d.b. of calcium, while in products with 30% of HW content, the level of calcium increased to 55.289 g/kg d.b. At the same time, there was a significant increase in the content of Mg and Na, while the level of K decreased. Increase in HW content also caused an increase in the levels of microelements. An increase was observed in the levels of Fe, Cu, Ni, Cr, and Zn (Table 4).

**Table 3:** Chemical composition and detergent fiber contents of the extrudates (% d.b.)

	Samples					
	HW5	HW10	HW15	HW20	HW25	HW30
Dry matter	91.12^c^	91.32^cb^	92.30^ba^	92.13^cba^	93.08^a^	93.09^a^
Protein	10.93^c^	11.06^cb^	11.04^cb^	11.43^ba^	11.54^a^	11.61^a^
Crude fat	3.74^d^	4.15^c^	4.46^b^	4.63^ab^	4.75^a^	4.82^a^
Ash	5.66^f^	9.54^e^	13.79^d^	17.98^c^	22.64^b^	27.18^a^
NDF	8.54^a^	8.38^ab^	8.38^ab^	8.18^b^	8.14^b^	8.13^b^
ADF	2.82^a^	2.61^a^	2.63^a^	2.67^a^	2.62^a^	2.60^a^
Hemicelluloses^a^	5.72^a^	5.77^a^	5.75^a^	5.51^a^	5.52^a^	5.53^a^
Cellulose^b^	2.27^ab^	2.06^ab^	2.08^ab^	1.98^ab^	1.89^ab^	1.79^b^
Lignin	0.55^b^	0.55^b^	0.55^b^	0.69^ab^	0.73^a^	0.81^a^

Mean values with different letters (a–f) in the same row are significantly different (Duncan test, *P* < 0.05); NDF – neutral detergent fiber; ADF – acid detergent fiber; ^a^Hemicelluloses = NDF–ADF; ^b^Cellulose = ADF–Lignin; hemicellulose and cellulose contents were calculated from replicates; HW – hatchery waste.

**Table 4:** Composition of macro- (g kg^−1^ d.b.) and microelements in the extrudates (mg kg^−1^ d.b.)

		Samples					
		HW5	HW10	HW15	HW20	HW25	HW30
Macroelements	K	1.129^a^	1.043^b^	0.958^c^	0.936^c^	0.915^c^	0.853^d^
	Ca	12.639^f^	20.676^e^	29.348^d^	34.605^c^	46.568^b^	55.289^a^
	Na	0.401^e^	0.504^d^	0.581^c^	0.643^bc^	0.682^ab^	0.731^a^
	Mg	1.546^c^	1.558^bc^	1.633^b^	1.730^a^	1.754^a^	1.780^a^
Microelements	Fe	20.474^f^	32.192^e^	40.277^d^	48.087^c^	55.041^b^	62.038^a^
	Cu	3.393^d^	4.117^c^	4.238^c^	4.512^b^	4.564^b^	4.829^a^
	Ni	1.990^e^	2.142^de^	2.260^dc^	2.317^c^	2.580^b^	2.793^a^
	Cr	0.988^ed^	0.961^e^	1.022^d^	1.161^c^	1.258^b^	1.350^a^
	Zn	25.048^e^	26.453^d^	27.696^c^	27.769^c^	28.202^b^	29.434^a^

Mean values with different letters (a–f) in the same row are significantly different (*P* < 0.05); HW – hatchery waste.

Compared to corn meal, HW is a material that is richer in proteins and fat (Table 1). The increase of its percentage share in extrudates from 5 to 30% caused an increase in the protein content from 10.93 to 11.61% d.b. (Table 3). There was also a significant increase in the content of crude fat (from 3.74 to 4.82% d.b.) (Table 3), while there was a decrease in the levels of NDF (Table 3) and TDF (Table 5). Upon increasing the content of the waste component, no significant changes were found in the levels of ADF and SDF, while there was a significant increase in the content of lignins. The content of TDF in the extrudates studied was higher compared to the level of NDF and varied from 8.21 to 11.01% d.b. for products with 30% and 5% content of the waste component, respectively (Table 5). The dominant fraction of dietary fiber, assayed with the enzymatic method, was the insoluble fraction (IDF) (Table 5).

**Table 5:** Composition of the enzymatic dietary fiber in the extrudates (% d.b.)

	Samples					
	HW5	HW10	HW15	HW20	HW25	HW30
IDF	8.35^a^	7.90^b^	7.37^c^	6.66^d^	5.87^e^	5.25^f^
SDF	2.66^a^	2.66^a^	2.73^a^	2.73^a^	2.73^a^	2.96^a^
TDF^a^	11.01^a^	10.56^ab^	10.10^b^	9.39^c^	8.60^d^	8.21^d^

Mean values with different letters (a–f) in the same row are significantly different (*P* < 0.05); TDF – total dietary fiber; IDF – insoluble dietary fiber; SDF – soluble dietary fiber; ^a^TDF = IDF + SDF; HW – hatchery waste.

The content and fractional composition of dietary fiber in feeds for animals have an effect on the rate of intestinal passage, digestion, and absorption of nutrients [36]. In this regard, a high importance is attributed to the soluble fraction of dietary fiber. Its increase leads to increased viscosity of the gastric and intestinal content and, in consequence, causes a reduction in the absorption of glucose and other nutrients [36]. This may cause an inhibition of the mass increase in animals. The content of the particular components of dietary fiber, such as lignins, cellulose, or hemicelluloses, is also important, which, in the case of various animals, can be digested to a varying degree and can produce varied energy effects [37]. The literature data report that HW contains minor amounts of dietary fiber [7,9]. Hence, with an increase in the content of HW in the extrudates a decrease was noted in the content of both NDF and TDF. The study demonstrated considerable differences in the content of dietary fiber in relation to the assay method employed. The NDF was lower than the TDF. Similar relationships were also noted by Kasprzak and Rzedzicki [38] and Sobota et al. [39].

## 4 Conclusions

The technology of twin-screw extrusion can find an application in the utilization of HW and responsible disposal of this kind of waste product in animal feed production. The addition of HW to corn meal in amounts not exceeding 30% and the application of blend moisture at the level of 28% and extrusion temperature of 120/140/180/180/130°C permitted a stable run of the process and to achieve products with moisture not exceeding 9%. An increase in the share of HW in the extrudates resulted in a higher content of crude fat, protein, and minerals, especially calcium. A higher content of HW in raw material resulted in decrease of the ER, PDI, WAI, and WSI in the extrudate.
